# Physicochemical characterizations of functional hybrid liposomal nanocarriers formed using photo-sensitive lipids

**DOI:** 10.1038/srep46257

**Published:** 2017-04-13

**Authors:** Sumit Kumar Pramanik, Patricia Losada-Pérez, Gunter Reekmans, Robert Carleer, Marc D’Olieslaeger, Dirk Vanderzande, Peter Adriaensens, Anitha Ethirajan

**Affiliations:** 1 Institute for Materials Research (IMO), Hasselt University, Wetenschapspark 1 and Agoralaan D, 3590 Diepenbeek, Belgium; 2 IMEC, associated lab IMOMEC, Wetenschapspark 1, 3590 Diepenbeek, Belgium

## Abstract

With recent advances in the field of diagnostics and theranostics, liposomal technology has secured a fortified position as a potential nanocarrier. Specifically, radiation/photo-sensitive liposomes containing photo-polymerizable cross-linking lipids are intriguing as they can impart the vesicles with highly interesting properties such as response to stimulus and improved shell stability. In this work, 1,2-bis(10,12-tricosadiynoyl)-sn-glycero-3-phosphoethanolamine (DTPE) is used as a photo-polymerizable lipid to form functional hybrid-liposomes as it can form intermolecular cross-linking through the diacetylenic groups. Hybrid-liposomes were formulated using mixtures of DTPE and saturated lipids of different chain lengths (dipalmitoylphosphatidylcholine (DPPC) and dimirystoilphosphatidylcholine (DMPC)) at different molar ratios. The physico-chemical characteristics of the liposomes has been studied before and after UV irradiation using a combination of techniques: DSC, QCM-D and solid-state NMR. The results signify the importance of a subtle modification in alkyl chain length on the phase behavior of the hybrid-liposomes and on the degree of crosslinking in the shell.

Over decades liposomes have emerged as versatile and promising nanocarrier platforms in the biomedical field as they can be used for encapsulating hydrophobic as well as hydrophilic payloads^1^. Despite offering several attractive qualities such as convenient surface functionality incorporation, carrier for wide range of cargo molecules (drugs, vaccines, imaging markers) and possibilities for various routes of administration, the mechanical stability and the inherent leaky nature of the liposomes hinders their potential in the nanomedicine field^2^,^3^. Therefore, improving the shell properties of liposomes and incorporation of controlled release with site-specific delivery aspects have stimulated lot of interests where design of nanocarriers with useful properties has been of great focus^4^,^5^,^6^,^7^. In this regard, efforts to safely transport drugs/other payloads to the target site by improving shell stability has been paralled by attempts to impart better control on release by rendering liposomes with properties such as response to stimulus or release on trigger^1^,^8^,^9^,^10^.

The properties of the liposomes depend on lipid composition, surface charge, size, and the method of preparation^11^,^12^. The ‘rigidity’ or ‘fluidity’ of the liposomes depends on the composition of the bilayer^13^,^14^. As compared to conventional liposomes, photo-polymerized vesicles (containing unsaturated phosphatidylcholine species) display enhanced mechanical stability and enhanced retention of lipophilic compounds^15^,^16^. The photo-polymerizable lipids in liposomes upon UV exposure are crosslinked and form polymeric chains which restrict the fluid nature of the membrane^8^. Although membrane fluidity of liposomes is an essential cell membrane characteristic for functional interaction with extracellular proteins, rigidity is in turn also important for delivery of drugs at a specific rate^17^,^18^. Generally the polymerization is related to rigidity which gives the membrane enhanced stability^19^. In this regard, polymer conjugation or crosslinking of lipids in the membrane can enhance the impermeability of the shell against leakage of active ingredients from the carriers. The latter aspect is very crucial in the design of nanocarriers for drug delivery. As an advantage for the crosslinking lipids, previously, Chen *et al*. with their liposomal formulation have shown that polymerized liposomes have significantly improved stability compared with conventional liposomes both *in vitro* and *in vivo*^20^. Although highly desired, design of such carriers is challenging as the membrane property is highly dependent on the used lipid structure and the liposomal formulation.

The intriguing responsiveness to light inherent to photo-sensitive lipids has made these materials highly interesting. On the one hand, stable liposomes can be formed by photo (UV) polymerization where covalent network between lipids chains can be formed and improved non-covalent interaction can be achieved^21^. On the other hand, photo-triggerable liposomes can be used for on-demand drug delivery where the drug is released from the liposome in a controlled manner by light triggering^22^. Alonso and co-workers have demonstrated that ultraviolet irradiation of diacetylenic liposomes exhibited improved size stability after a period of 30 days at 4 °C and also interfacial membrane characteristics were found to be altered after polymerization^23^. The controlled release of the encapsulated drug by photo-trigger has been previously demonstrated for liposomes containing mixtures of photopolymerizable lipids and saturated lipids^16^,^24^. Although the applicability of this class of materials has stimulated a lot of interest lately, only a limited number of studies exists on liposomes formulated using photo-sensitive lipids that elucidates their physical and chemical properties upon exposure to irradiation. So far, the miscibility and phase behavior of polymerizable lipid mixtures is restricted to diacetylenic-based lipid dispersions using differential scanning calorimetry (DSC), electron spin resonance (ESR) and to SorbPC lipids using nuclear magnetic resonance (NMR)^25^,^26^,^27^.

In order to improve the understanding of these systems we have structurally characterized non-polymerized and polymerized hybrid liposomes containing photo-polymerizable lipids. To this end, we have studied binary mixtures consisting of a saturated phospholipid and 1,2-bis(10,12-tricosadiynoyl)-sn-glycero-3-phosphoethanolamine (DTPE) as photo-polymerizable lipid. We have chosen two saturated phospholipids differing in two ethyl groups, namely, dipalmitoylphosphatidylcholine (DPPC) and dimirystoilphosphatidylcholine (DMPC). The chemical structures of the above mentioned lipids are shown in Fig. 1. The scheme of the photo-polymerization reaction is shown in Figure S1 (Supplementary Information).

Liposomes were prepared using different stoichiometric ratios of DTPE and DPPC/DMPC and their phase behavior was systematically characterized before and after UV irradiation. DSC was used to study the thermal properties as the latter is extremely sensitive to lipid-lipid interaction and packing. The mechanical properties before and after UV-exposure were monitored by Quartz crystal microbalance with dissipation monitoring (QCM-D), which provides the information about the viscosity of the two bilayer phases, gel and fluid. The molecular mobility as a function of temperature and chemical changes were studied by Solid-state ^1^H-wideline NMR measurements, which gives independent information about the structure of the two bilayer phases before and after UV-exposure.

## Methods

### Materials

DMPC, DPPC, and DTPE were purchased from Avanti Polar Lipids (Alabaster, AL). Spectroscopic grade chloroform (assay 99.3% stabilized with about 0.6% ethanol) was obtained from VWR. HEPES buffer (pH 7.4) consisting of 10 mM HEPES from Alfa Aesar, (assay 99%) and 150 mM NaCl from Sigma-Aldrich (assay ≥99.5%) was used for hydration of the dried lipid film.

### Liposome preparation

The lipid or lipid mixture was first dissolved in chloroform in a scintillation vial, blown dry with a mild flow of nitrogen and dried under overnight vacuum. The lipid film is then hydrated to 2 mg/mL with HEPES buffer, swirled and sonicated in a VWR ultrasonic cleaner for 20 s to detach the lipid, as well as to promote its dispersion. The vial is then incubated in an oven for 2 h at temperatures above the melting point of both phospholipids, DMPC was incubated at 44 °C while DPPC at 61 °C. An extruder purchased from Avanti Polar Lipids (USA) was used to form small unilamellar liposomes. The lipid or lipid mixture dispersion in HEPES buffer is extruded for 25 times through a polycarbonate membrane with pore size of 100 nm to form liposomes of approximately ~ 125 ± 40 nm.

For the irradiation of the liposomes, an OMNICURE Series 1000 lamp system with four-arm setting was used as a UV light source. The system was equipped with a 100 W high pressure mercury vapor short arc lamp. Using an iris setting of 50%, the sample was irradiated for 20 min. While one of the UV lamp arms illuminated the sample from the top, the other three arms were used for illumination from different sides of the vial.

### Dynamic light scattering (DLS)

The average size and size distribution (polydispersities) of the liposomes in HEPES buffer (pH 7.4) consisting of 10 mM HEPES and 150 mM NaCl were measured at 20 °C by dynamic light scattering using a Brookhaven instruments Zetapals. 4 cycles of one minute were performed to obtain size and polydispersity from light scattering intensity data.

Polydispersity has no units. It is close to zero (0.000 to 0.020) for monodisperse or nearly monodisperse samples, small (0.020 to 0.080) for narrow distributions, and larger for broader distributions.

### Differential Scanning Calorimetry (DSC)

The thermal analysis of aqueous dispersions of pure lipids and lipid mixtures were recorded by a differential scanning calorimeter DSC Q200 by TA Instruments with a heating and cooling rate of 0.2 °C/min. A 5 μl aliquot of lipid or lipid mixture dispersion was placed in a hermetically sealed aluminum pan.

### Quartz Crystal Microbalance with Dissipation (QCM-D)

In QCM-D an AC voltage is pulsed across an AT-cut piezoelectric quartz crystal, causing it to oscillate in shear mode at its resonant frequency f, which is recorded in real time, and depends on the total oscillating mass and the intrinsic properties of the quartz crystal. The damping of the shear wave is also recorded as the dissipation factor (D). Both f and D give information about the viscoelastic properties of the film^28^. We have used QCM-D on a Q-sense E4 instrument (Gothenborg, Sweden) which enables heating or cooling temperature scans from 15 to 50 °C. We have chosen six resonant frequencies (overtones, n = 3^rd^ to 11^th^).

AT-cut quartz crystals with gold coated sensors (diameter 14 mm, thickness 0.3 mm, surface roughness 3 nm and resonant frequency 4.95 MHz) have been used. The gold-coated quartz sensors were first cleaned with a 5:1:1 mixture of Milli-Q water, ammonia and hydrogen peroxide at 80 °C and then with acetone and dried under N_2_ flow. Before using they were exposed to UV-ozone for 15 min using a Digital PSD series system from Novascan.

First, a baseline in HEPES buffer was established and then liposomes were injected over the gold-coated sensor with a flow rate of 50.10 μL/min for 20 min after which the pump was stopped. Temperature scans with alternating heating and cooling were performed at a rate of 0.2 °C/min, maintaining 60 min of stabilization between successive ramps.

### Nuclear Magnetic Resonance (NMR)

Solid-state ^1^H-wideline NMR measurements were carried out on a Varian Inova 400 spectrometer in a dedicated wide-line probe equipped with a 5 mm coil using solid echo methods. The samples (50 mg) were placed in 5 mm glass tubes tightly closed with teflon stoppers.

The T_2H_ (spin-spin relaxation) decay times were derived from on-resonance Free Induction Decays (FID’s - 48 accumulations) obtained by the solid echo technique (90°_x’_ - t_se_ - 90°_y’_ - t_se_ - acquire) to overcome the effect of the dead-time of the receiver. The 90° pulse length t_90_ was set to 1.5 μs and spectra were recorded with a spectral width of 2 MHz (0.5 μs dwell time) allowing an accurate determination of the echo maximum. The echo maximum is formed at τ = (3t_90_/2 + 2t_se_) = 7 μs and this time point is calibrated to time zero. A preparation delay of 5 times the T_1H_ decay time was respected to obtain quantitative results.

The intensity was analyzed as a function of the acquisition time t according to the following equations:

At low temperatures (below 70 °C for DPPC + 20% DTPE, 65 °C for UV-exposed DPPC + 20% DTPE and DMPC + 20% DTPE and 60 °C for UV-exposed DMPC + 20% DTPE), the FID curves were analysed with the sum of a Gaussian and Lorentzian shape function:


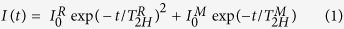


At higher temperatures the FID curves of the samples containing DPPC + 20% DTPE, UV-exposed DPPC + 20% DTPE and UV-exposed DMPC + 20% DTPE were analysed with the sum of a Gaussian and two Lorentzian shape functions:


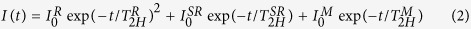


For the sample containing DMPC + 20% DTPE at higher temperatures, the FID curves were analysed with the sum of a Weibull (stretched exponential) and Lorentzian shape function:


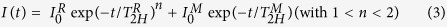


where the superscripts 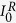, 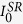 and 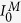
refer to the rigid proton fraction (short decay time), semi-rigid proton fraction (intermediate decay time) and mobile proton fraction (long decay time), respectively.

All experimental data were analyzed using a non-linear least-squares fit (Levenberg-Marquardt algorithm). An overview regarding the FID analysis procedure and shape functions used to fit the experimental FID’s can be found in literature^29^,^30^.

## Results and Discussion

We present a systematic study of different types of lipid structures, namely unilamellar lipid dispersions, solid-supported unilamellar vesicle layers and lyophilized unilamellar vesicle dispersions, which have been measured by DSC, QCM-D and Solid state NMR, respectively. We investigate the effect of the photo-polymerizable lipid on the phase behavior of saturated phosopholipids and study the difference before and after UV-exposure.

The average diameter and polydispersity index of the samples were determined by DLS and are presented in Table 1. The average size of the liposomes ranges between 125–165 nm and the polydispersity index increases with increasing amount of the photo-polymerizable DTPE lipid. The presence of the unsaturated lipids poses stress on the liposomal structure and thus increasing concentration of the unsaturated lipid leads to a decrease of the stability of the liposomes. This is in agreement with previous studies, where unstable liposomes were formed above 20 mol% of unsaturated lipids^16^,^27^. Therefore only liposomes containing up to a maximum of 20 mol% of DTPE were used here. The liposomes with 10 and 20 mol% DTPE were not affected by UV treatment, since the size as well as the PDI do not show much variation after UV exposure.

**Figure 2:**
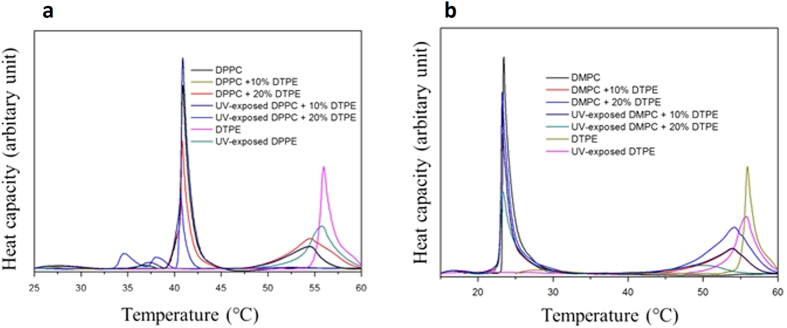
DSC thermograms (heating cycle) of different samples. (**a**) DPPC and (**b**) DMPC vesicles containing 0, 10 and 20 mol % DTPE.

DMPC and DPPC vesicles undergo a sharp transition (main phase transition) at 23.5 °C and 40.9 °C upon heating, respectively (see Fig. 2), in accordance to previous calorimetric measurements^19^,^31^,^32^,^33^. For both systems, addition of DTPE results in a shift of the melting temperature of the pure lipid to lower temperature, in addition to peak broadening. Simultaneously, a higher temperature transition arises around the melting of pure DTPE (50–60 °C). With increasing amount of DMPC or DPPC, the height of the peak of DTPE decreases, becomes broader and is located at a slightly lower temperature as compared to the one of pure DTPE. This denotes the existence of DTPE-rich domains coexisting with DMPC or DPPC-rich domains^27^.

The relative contribution of the two main components is observed and no major change occurs for the main phase transition of the pure lipids. The melting of the DMPC-rich domains barely changes from the melting temperature of pure DMPC and the same takes place for the DPPC lipid. After UV-exposure of samples with 20 mol % DTPE, the main phase transition of both DPPC and DMPC is broadened downwards. For DPPC/DTPE a peculiar shape with two maxima is observed in proximity of DPPC main phase transition peak and concomitantly, the phase transition of DTPE around 55 °C is no longer detected. On the contrary, for the DMPC/DTPE system, the phase transition of DTPE is shifted to lower temperature (50 °C). This gives the first indication that the effects of UV exposure in both the systems are different and the interaction of DTPE moieties with the saturated lipids are distinct. No characteristic difference was observed after UV-exposure for samples containing 10% of DTPE. This can be attributed to the low concentration of the photo-polymerizable lipids. For this reason, we have performed all further experiments with samples containing 20% of DTPE. The formation of the adsorbed lipid layer on the gold coated quartz substrates depends on the composition of the liposomes and the temperature of deposition^34^,^35^. The liposomes were adsorbed below the main transition temperature of the pure phospholipids (DMPC − DTPE systems at 16 °C and DPPC − DTPE systems at 20 °C)^36^,^37^. Liposome adsorption takes place by reaching a stable plateau at large Δf/n and ΔD values for all the studied mixtures, where n stands for the overtone number. Moreover, the different overtones are not overlapping, indicating the formation of a non-rigid liposome layer^36^,^38^,^39^. Figure S2 (Supplementary information) shows a typical example of an intact solid-supported vesicle layer for DPPC (a), DPPC + 20% DTPE (b), DMPC (c) and DMPC + 20% DTPE (d).

**Figure 3:**
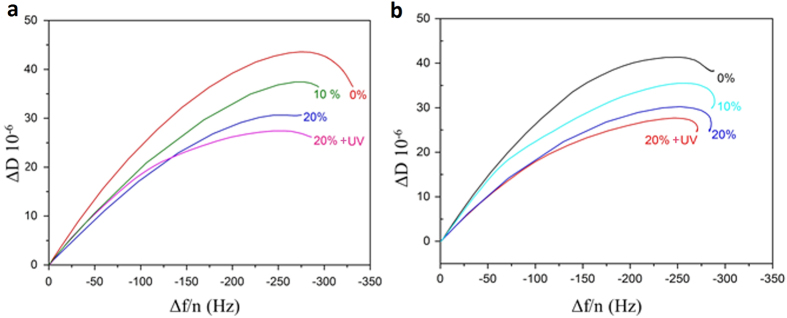
ΔD − Δf/n plots for the 7th overtone. (**a**) DPPC + DTPE and (**b**) DMPC + DTPE systems.

In order to assess the influence of DTPE on the formation of liposome layers, ΔD − Δf/n plots are displayed in Fig. 3. Although samples containing 10 mol % of DTPE did not show characteristic change after UV exposure, we show here samples with 10 mol % DTPE for both systems (DPPC and DMPC) for the sake of understanding the influence of DTPE addition. In these plots time is eliminated as an explicit adsorption parameter and they provide insights into the viscoelastic nature of the formed adlayer. The positive slope of the ΔD − Δf/n curves denotes progressive liposome adsorption reaching final ΔD and Δf/n values. While the final values of Δf/n slightly change with DTPE, ΔD decreases clearly with increasing amount of DTPE. This indicates that diacetylenic groups in DTPE induce more rigid liposome layers. This applies to both DMPC and DPPC + DTPE bilayer mixtures.

After UV exposure an even larger ΔD change is observed. The diacetylenic groups of DPTE are highly reactive upon UV irradiation and form covalently linked lipid chains in the bilayer which makes the liposomes even more rigid, in agreement, as we shall see with solid-state wideline NMR measurements.

Shear viscosity temperature profiles were calculated using a Voigt-based viscoelastic model^40^. Overtones 3^rd^ to 11^th^ were fitted using the software Qtools (Q-sense AB, Sweden) keeping as fixed parameters the density of the fluid 1.0 g cm^−3^, the viscosity of the fluid 0.001 Pa s and the density of the lipid layer 1.06 g cm^−3^ 37.

**Figure 4:**
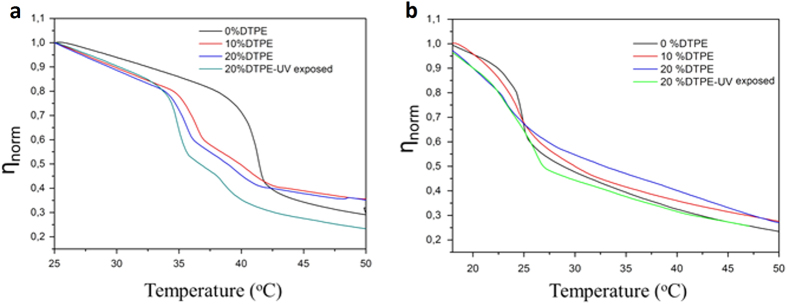
Temperature dependence of η_norm_. (**a**) DPPC + DTPE mixtures, (**b**) DMPC + DTPE mixtures.

The obtained shear viscosity values are effective values since the model assumes a homogeneous layer and our layers were not completely homogeneous, especially DTPE containing systems whose polydispersity was greatly enhanced with increasing amount of DTPE. Figure 4 shows the temperature dependence of the normalized effective viscosity 
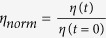
 for all the studied systems upon heating. For pure DMPC and DPPC systems, the transition from a more viscous (gel) to a less viscous (liquid) state is reflected as a steep decrease in *η (T*).

The narrow transition range for both DMPC and DPPC systems reflects the cooperative character of their main transition and falls within the range observed in previous calorimetric measurements^31^,^41^,^42^. For mixtures containing DTPE the onset of the transition is shifted to lower temperature and the slope of the shear viscosity decrease is shallower. The transition takes place in a wider temperature range denoting the loss of cooperativity in the presence of DTPE. A comparison of the obtained η_norm_ (*T*) profiles with the thermograms obtained by DSC is unfortunately not possible due to the temperature limitation of the QCM-D device (T_max_ = 50 °C). Moreover, the transition temperatures cannot be directly compared since in QCM-D we are dealing with solid-supported unilamellar vesicle layers, whose melting will be affected by the presence of the solid support, instead of multilamellar vesicle dispersions in bulk as measured by DSC^43^. Nevertheless, qualitative analogies take place and can be established for both DMPC and DPPC mixtures. The melting temperature of DPPC decreases more as compared to that of DMPC in the presence of the same amount of photo-polymerizable lipid. This indicates that the chain mobility in DPPC is more increased than in DMPC. In other words, DTPE significantly weakens the intermolecular van der Waals forces between DPPC alkyl chains as compared to DMPC. In addition, upon UV-irradiation, DPPC + 20% DTPE mixtures exhibit a peculiar shape in the vicinity of the melting temperature of pure DPPC as also observed by DSC.

The solid-state wideline NMR measurements were accomplished on freeze dried liposomes containing 20% of DTPE which, according to DSC and QCM-D data, results in characteristic changes especially in the DPPC/DTPE system under UV irradiation. Solid-state ^1^H-wideline NMR is a sensitive technique by which the fragmental chain mobilities can be straightforwardly probed on the molecular level by an analysis of the T_2H_ relaxation behavior^44^,^45^,^46^. T_2H_ relaxation (or proton spin-spin relaxation) is the process describing the loss of proton transverse magnetization after a short 90° proton pulse due to proton spin-spin interactions. The faster the (fragmental) chain mobility, the more these dipole-dipole interactions become averaged out, resulting in longer T_2H_ decay times (slower loss of transverse magnetization or less efficient relaxation). In other words, the more rigid the chains, the stronger the proton dipole-dipole interactions and the shorter the T_2H_ decay time (faster loss of transverse magnetization). The T_2H_ relaxation behavior can be obtained by curve fitting analysis of the on-resonance solid-echo FID (free induction decay) of the specimen. If more frequency distributions of chain motion are present, more T_2H_ decay times are needed to describe the FID. In that case, the longest decay time represents the fraction of protons in mobile chain fragments, the shortest decay time represents the fraction of protons in rigid chain fragments and the intermediate decay time (if present) represents the protons in chain fragments with intermediate mobility. So in general, the T_2H_ relaxation behavior is a probe to describe and quantify the phase morphology based on differences in molecular chain mobility.

For DMPC as well as DPPC with 20% DTPE before and after UV exposure, the FID’s obtained from measurements below 60–70 °C had to be analyzed with a Gaussian and Lorentzian (exponential) shape function, representing the rigid and mobile liposome chain fragments, respectively. At higher measuring temperatures, an additional Lorentzian function had to be added, representing semi-rigid chain fragments. For the DMPC/DTPE system however, the FID curves obtained at and above 65 °C had to be analyzed with a Weibullian and Lorentzian shape function.

**Figure 5:**
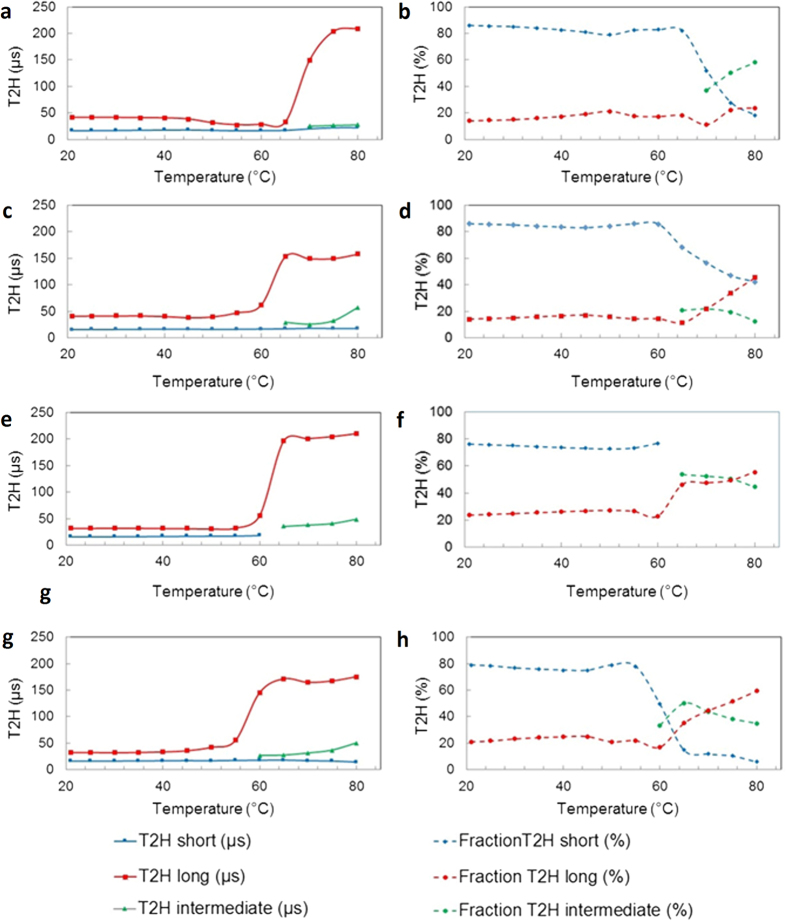
Solid-state wide line NMR measurements. (**a** and **b**) DPPC + 20% DTPE, (**c** and **d**) UV exposed DPPC + 20% DTPE, (**e** and **f**) DMPC + 20% DTPE and (**g** and **h**) UV exposed DMPC + 20% DTPE.

Figure 5a and b show the evolution of the T_2H_ decay times and their corresponding fractions as a function of temperature for the DPPC/DTPE liposomes. The rigid proton fraction (blue dotted line), representing chain fragments that are immobilized by intensive intra- and interchain van der Waals (vdW) interactions between the long C16 side chains, remains around 80% up to 65 °C. The T_2H_ decay time (i.e. mobility) of protons in these rigid chain fragments (blue line) remains very short, around 20 μs, even at 80 °C. The mobile proton fraction (red dotted line), representing mobile chain fragments (e.g. chain ends, polar phosphate head groups), remains quasi constant at 20% up to 80 °C. The T_2H_ decay time of these chain fragments (red line) on the other hand increases strongly from about 30–40 μs below 65 °C to more than 200 μs at 80 °C. A clear switch in chain mobility starts to appear above 65 °C. At 70 °C, the rigid fraction strongly drops from 80% to about 50% while a new semi-rigid fraction (green dotted line) appears. The transition results from the melting process, which takes place at a higher temperature as compared to the gel-liquid transition measured by DSC and QCM-D. Further increase of the temperature to 80 °C results in a further drop of the rigid fraction to about 20% and an increase of the semi-rigid fraction to about 60%.

The T_2H_ relaxation characteristics of the UV exposed DPPC/DTPE liposomes show some major differences at high temperatures. The main changes, as demonstrated in Fig. 5c and d, are observed for the fraction of rigid chain fragments (blue dotted line), the T_2H_ decay time (red line) and fraction (red dotted line) of the mobile chain fragments. The loss of rigid chain fragments is clearly much smaller as compared to the untreated liposomes (remaining 40% versus 20% at 80 °C). This is a first indication for UV induced cross-linking of the system. Moreover, despite that the fraction of mobile chain fragments increases due to partial disruption of vdW interactions (45% versus 20% at 80 °C), these mobile chain fragments are less mobile (T_2H_ decay time of 160 μs versus more than 200 μs for the untreated liposomes at 80 °C). These results confirm the cross-linking of these UV exposed DPPC/DTPE liposomes, as already observed via the melting behavior in DSC and loss of cooperativity of the shear viscosity change in QCM-D.

Figure 5e and f show the evolution of the T_2H_ decay times and corresponding fractions as a function of temperature for liposomes composed of DMPC/DTPE. Up to 60 °C, the fraction of rigid chain fragments is high and quasi constant at 75%. Above 60 °C, this fraction strongly drops to about 50–55% due to the melting transition. Remark that the FID can already be fitted here by means of the sum of a Weibullian and Lorentzian shape function (instead of a Gaussian and two Lorentzian functions in the case of DPPC based liposomes). As a consequence of the weaker vdW interactions between the shorter C14 chains, the molecular mobility of the system in general is higher, somewhat comparable to rubbery systems. Increasing the temperature above 60 °C results also in a strong increase of the mobility of the mobile chains (and so long T_2H_ decay time; see red line) from about 30 μs below 60 °C to more than 200 μs at 80 °C.

Starting from the melting transition, the evolution of the fraction of the rigid chain fragments (blue dotted curve) is completely different for the UV exposed DMPC/DTPE liposomes (Fig. 5g and h), as compared to the cross-linked DPPC/DTPE liposomes. At 65 °C, only a very small fraction of rigid chains remain (5–10%). This behavior is similar to DPPC/DTPE system before cross-linking (Fig. 5a and b) except that it already takes place at a lower temperature 65 °C due to the weaker vdW interactions between the shorter C14 chains. The less vdW interactions between DMPC molecules can possibly allow for relatively easy incorporation of DTPE molecules into the DMPC bilayer, thereby leading to a better distribution of the polymerizable lipids in the shell. In all likelihood, the latter manifests in to limited degree of crosslinking in the shell, as compared to DPPC/DTPE hybrid liposomes.

In summary, we have carried out a detailed study of binary mixtures of lipids for the formulation of hybrid liposomes - containing DTPE, a photo-polymerizable lipid used for functional nanocarriers - combining DSC, QCM-D and NMR spectroscopy. The combination of several techniques has successfully shed light on the physico-chemical characteristics of the hybrid liposomes. Hybrid liposomes were prepared using different lipid ratios and subjected to UV-exposure in order to elucidate the change in membrane properties after irradiation. Our findings show that the melting transitions in DPPC and DMPC are not significantly affected by UV irradiation. However, the study clearly indicates that the effects of UV exposure in DPPC/DTPE systems are different and the interactions of DTPE moieties with the two saturated lipids are distinct. QCM-D shows a decrease of layer dissipation upon increase in DTPE concentration, indicating that the layers get more rigid as the photo-polymerizable lipid is added. Independent solid-state wideline NMR measurements of the T_2H_ relaxation, a probe to describe and quantify the morphology based on differences in molecular chain mobility, confirm that the both types of hybrid liposomes become cross-linked upon UV irradiation. However, as compared to DTPE-DPPC based liposomes the extent of crosslinking for DTPE-DMPC based liposomes is less pronounced. This can possibly be attributed to the limited degree of crosslinking between the DTPE molecules as a consequence of a better distribution of DTPE molecules between the DMPC molecules, which display weaker vdW interactions than DPPC ones. This reflects how a subtle modification in chain length can induce significant changes in the phase behavior of the hybrid liposomes and on the degree of crosslinking in the shell due to the photopolymerizable lipids. The present discussions on structural characteristics and system understanding will have implications in promoting usage of mixtures containing polymerizable lipids when designing new systems with potential applications as nanocarriers for new drug delivery system. Studying in detail the trigger responsiveness of the photosensitive liposomes and the incorporation of lipids with suitable functional end groups for conjugation of (bio-)molecules can lead to the development of advanced nanocarriers with useful functionalities enabling target specific release of therapeutics for *in vivo* drug delivery applications.

## Additional Information

**How to cite this article**: Kumar Pramanik, S. *et al*. Physicochemical characterizations of functional hybrid liposomal nanocarriers formed using photo-sensitive lipids. *Sci. Rep.* **7**, 46257; doi: 10.1038/srep46257 (2017).

**Publisher's note:** Springer Nature remains neutral with regard to jurisdictional claims in published maps and institutional affiliations.

## Supplementary Material

Supplementary Information

## Figures and Tables

**Figure 1:**
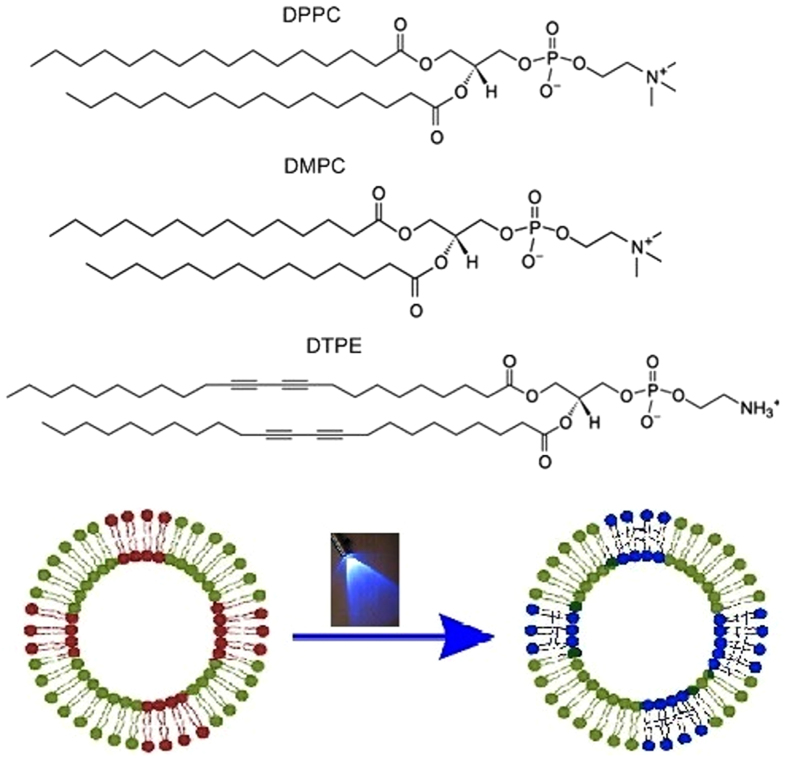
Chemical structure of DPPC, DMPC and DTPE. Schematic representation of photo-polymerization upon UV-exposure.

**Table 1:** Average Diameters (d) and Polydispersities of the Samples under Study.

composition	d (nm)	polydispersity
DPPC	126	0.05
DPPC + 10% DTPE	141	0.28
UV-exposed -DPPC + 10% DTPE	139	0.31
DPPC + 20% DTPE	160	0.33
UV-exposed -DPPC + 20% DTPE	163	0.35
DMPC	125	0.03
DMPC + 10% DTPE	137	0.15
UV-exposed -DMPC + 10% DTPE	135	0.19
DMPC + 20% DTPE	151	0.29
UV-exposed -DMPC + 20% DTPE	147	0.32

Percentages are given in mol%.
