# Supplementary material for: Physicochemical characterizations of functional hybrid liposomal nanocarriers formed using photo-sensitive lipids
Source: Sci Rep. 2017 Apr 13;7:46257. doi: 10.1038/srep46257 (PMC5390264; doi:10.1038/srep46257)
Supplement: Supplementary Information [file srep46257-s1.pdf]

Supplementary Information

Physicochemical characterizations of functional hybrid liposomal nanocarriers formed using photo-sensitive lipids

Sumit Kumar Pramanik[a,b], Patricia Losada-Pérez[a,b], Gunter Reekmans[a,b], Robert Carleer[a,b], Marc D'Olieslaeger[a,b], Dirk Vanderzande[a,b], Peter Adriaensens[a,b], and Anitha Ethirajan*[a,b]

[a] *Institute for Materials Research (IMO), Hasselt University, Wetenschapspark 1 and Agoralaan D, 3590 Diepenbeek, Belgium*

[b] *IMEC, associated lab IMOMEC, Wetenschapspark 1, 3590 Diepenbeek, Belgium*

Email: anitha.ethirajan@uhasselt.be

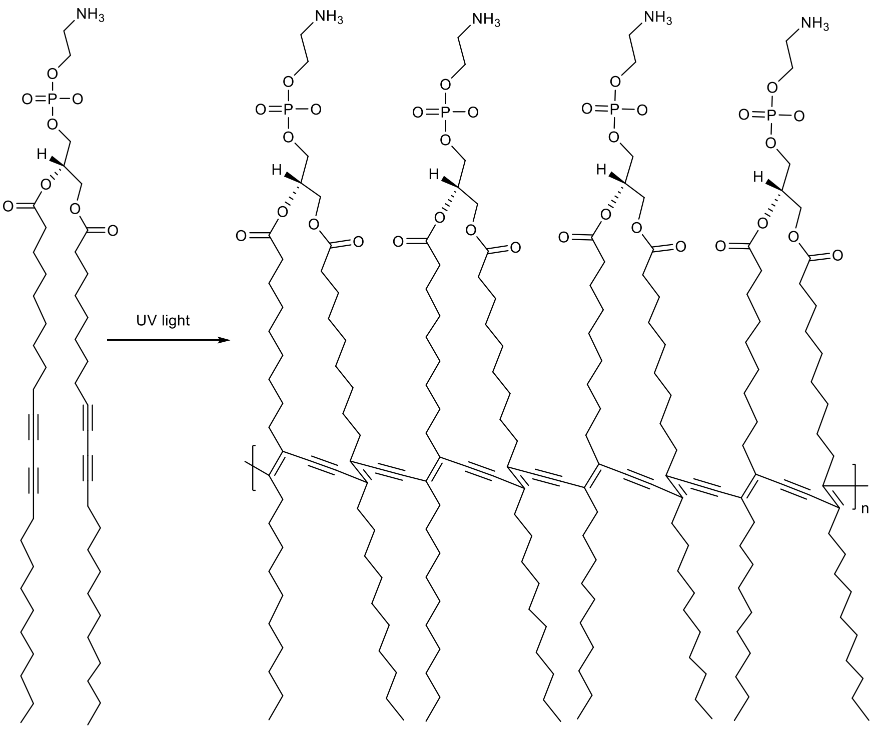

Figure S1: Scheme depicting the photo-polymerization reaction.

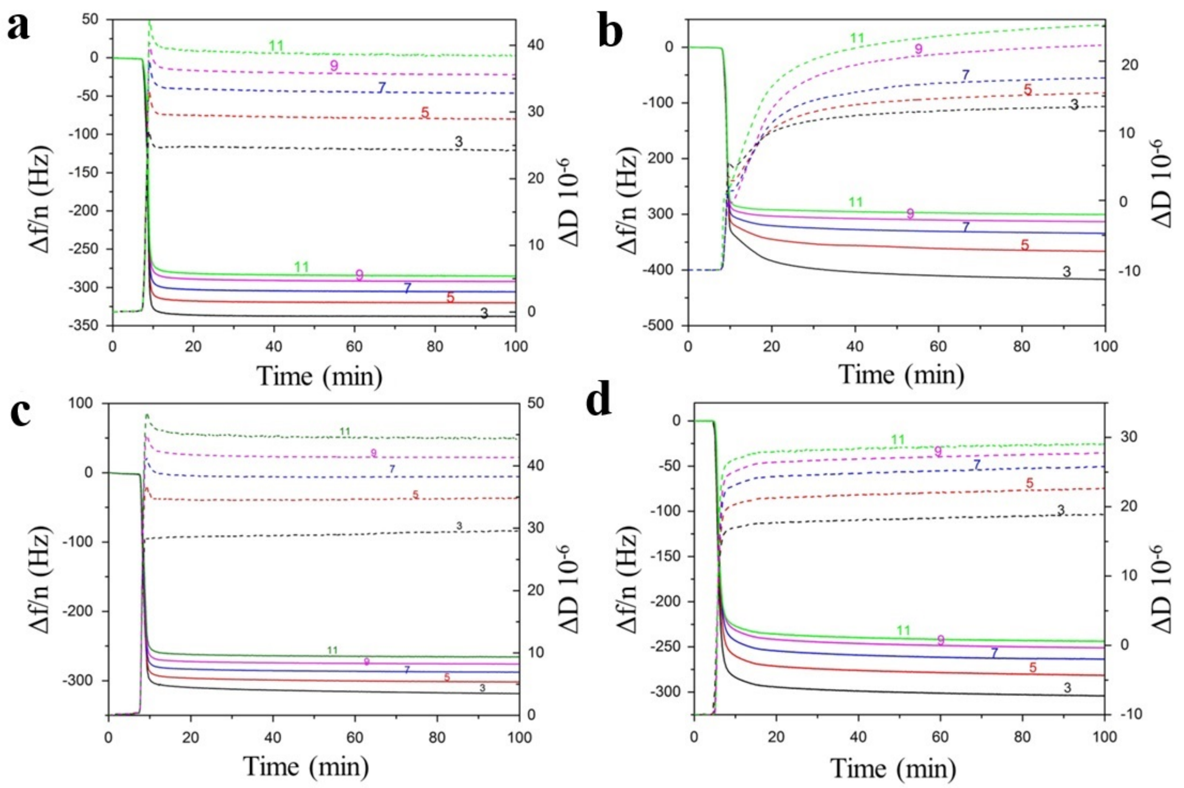

Figure S2: Time evolution of Δf/n (solid lines) and ΔD (dashed lines) of different overtones during a QCM-D experiment of (a) DPPC and (b) DPPC + 20 % DTPE liposome adsorption at 20 °C and (c) DMPC and (d) DMPC + 20 % DTPE liposome adsorption at 16 °C on gold- coated quartz sensor.
